# The eye in dengue fever, a rarely appreciated aspect of dengue expanded syndrome: a case report

**DOI:** 10.1186/s13256-019-2189-2

**Published:** 2019-08-29

**Authors:** Jevon Yudhishdran, Isurujith Kongala Liyanage, Mitrakrishnan Rayno Navinan, Sandamalee Herath, Danushka Withanage, Sivakumar Jeyalakshmy, Aruna Kulatunga

**Affiliations:** 1 National Hospital of Sri Lanka, Colombo, Sri Lanka; 2 Department of Clinical Pharmacology, Faculty of Medical Sciences, University of Sri Jayewardenepura, Nugegoda, Sri Lanka; 3 Colombo South Teaching Hospital, Dehiwala-Mount Lavinia, Sri Lanka

**Keywords:** Dengue, Dengue fever, Dengue retinopathy, Dengue maculopathy, Expanded dengue syndrome, Foveolitis, Central serous chorioretinopathy

## Abstract

**Background:**

Dengue fever is a mosquito-borne illness prevalent mainly in the tropics. It is feared for causing the dengue hemorrhagic spectrum of the disease leading to significant morbidity and mortality. Its rarer manifestations are categorized as the expanded dengue syndrome, and though being recognized, they are not fully appreciated and understood. The involvement of the eye in dengue fever is one such phenomenon.

**Case presentation:**

A 27-year-old South-Asian woman presented on day 2 of dengue fever, without capillary leakage, for further management. Despite developing hepatitis, she had an otherwise uncomplicated progression of the illness because she did not develop capillary leakage. On day 8 of the illness, she had the lowest platelet count and developed bilateral blurred vision. Examination revealed that only gross movements were detected in the left eye, and the right eye had a visual acuity of 6/9. She was diagnosed with foveolitis in the right eye and central serous chorioretinopathy in the left eye, along with hemorrhages in both eyes. These were confirmed by funduscopy, fluorescein angiography, optical coherence tomography, and macular scans. She received systemic and intravitreal steroids and was assessed regularly. After 6 months of observation, her visual acuity was 6/6 in the right eye and 6/9 in the left eye, which remained the same thereafter.

**Discussion:**

The exact mechanism of eye involvement in dengue viral infection is poorly understood. Multiple causes have been suspected and include viral factors, immune mediation, capillary leakage, stress, and hemorrhage. Eye involvement is classically seen at the lowest platelet count and when the count begins to rise. Though symptoms are nonpathognomonic, blurring of vision is the commonest complaint, but the range of presentation is extensive and variable. Ophthalmological assessment and funduscopy are very useful in addition to advanced assessments. There is no clear consensus on management; suggestions range from conservative care to aggressive steroid therapy with immune modulation and even ophthalmological intervention. Recovery can be full or partial with a variable time scale.

**Conclusion:**

The extensive spectrum of possible visual symptoms should prompt the clinician to suspect any visual complaint as potential dengue eye involvement. Guided studies and screening are needed to better understand the true incidence of eye involvement in dengue fever.

## Background

Dengue fever (DF) is a mosquito-borne disease with extensive worldwide reach [1]. Because urbanization promotes its spread, it is not surprising that it is found endemically in more than 100 countries globally [2]. Fever remains at the core of its numerous and varied presentations. Its progression to dengue hemorrhagic fever (DHF) should be managed promptly; otherwise, it leads to deadly complications. With growing vigilance, previously known but less appreciated atypical presentations such as neurological, gastrointestinal, respiratory, cardiac, renal, and eye involvement are gaining greater recognition as an extended spectrum of its manifestations, classified as expanded dengue syndrome [3]. Within this spectrum, the severe organ involvement can cause significant morbidity, which may become permanent or by itself result even in death. Though international and regional guidelines have begun to incorporate these varied presentations, there is still a lack of understanding of these guidelines as pathways of management, especially in management of complications.

The eye is not commonly involved in dengue viral infection, and patients with this presentation experience significant distress and pose an additional challenge to the physician. Furthermore, complications involving the eye can outlast the short-lived dengue viral infection [4]. In this case report, we describe a patient with dengue viral infection who developed ocular complications (for example, retinopathy) with residual deficit attributable to the disease, and we stress the importance of appreciating the potential rarer and less recognized complications that can occur due to dengue viral infection which can have a permanent impact.

## Case presentation

**Fig. 1:**
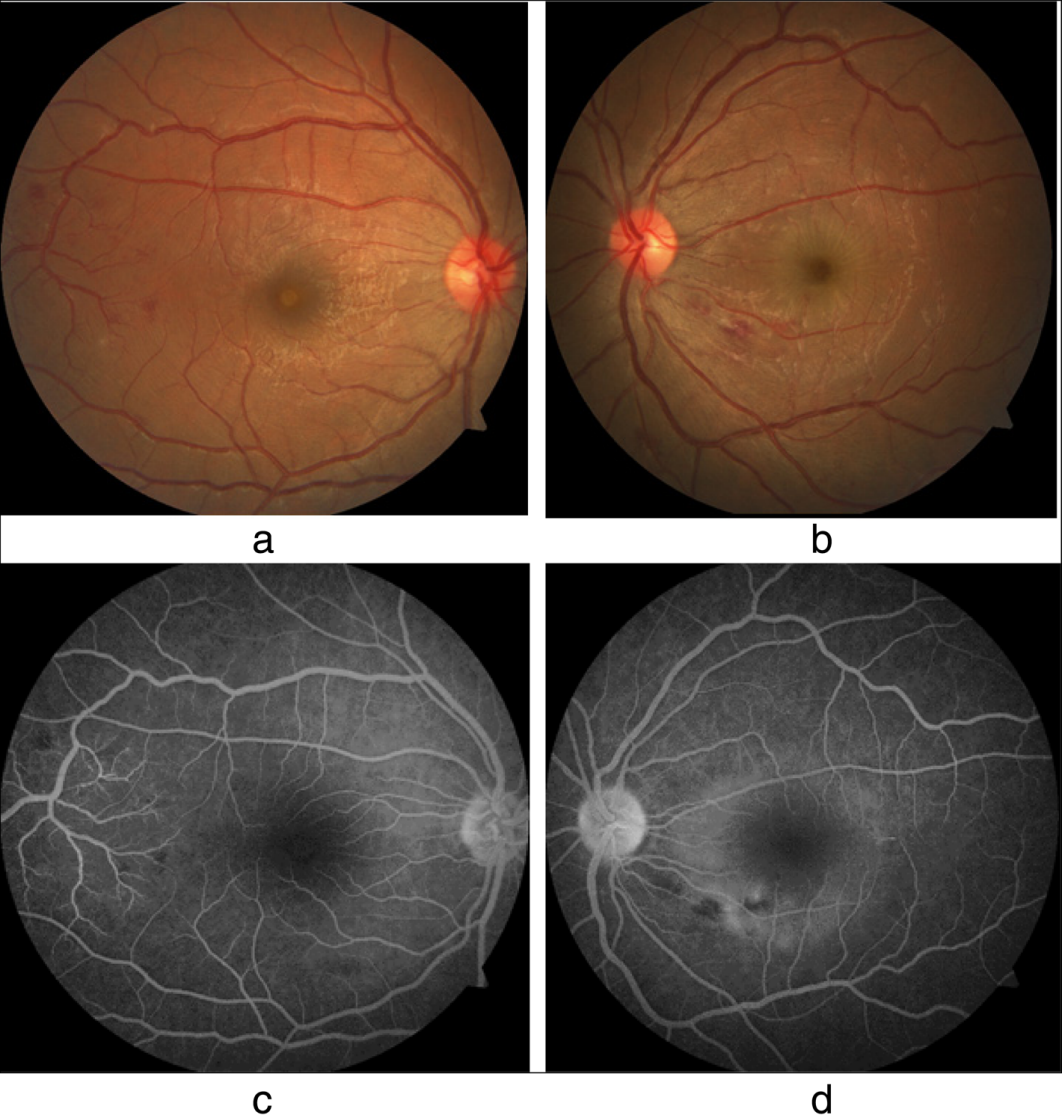
**a** and **b** are photo of both fundi. **a** is that of the right eye. The fovea (indicated by circle) appears relatively pale compared to that of what’s normal which is typical of dengue foveolitis. Additionally in the outer rim- temporally indicated by the arrow there are signs if resolving haemorrhages. **b** is that of the left eye. The faint circular light reflexion (indicated by the circle) centering around the fovea. This is the margin of retinal pigment epithelium (RPE) elevation. This demarcates the area of central serous chorioretinopathy (CSCR). Haemorrgahes are also observed. **c** and **d** are fourescein angiogras of both the right and left eye respectively. In **d**, the left eye the center of the macula and fovea is dark. There is a mild fluorecscnce of the fovea. The white halo clearly defines where the where the sub-retinal pigmental epithelium (RPE) fluid is present

**Fig. 2:**
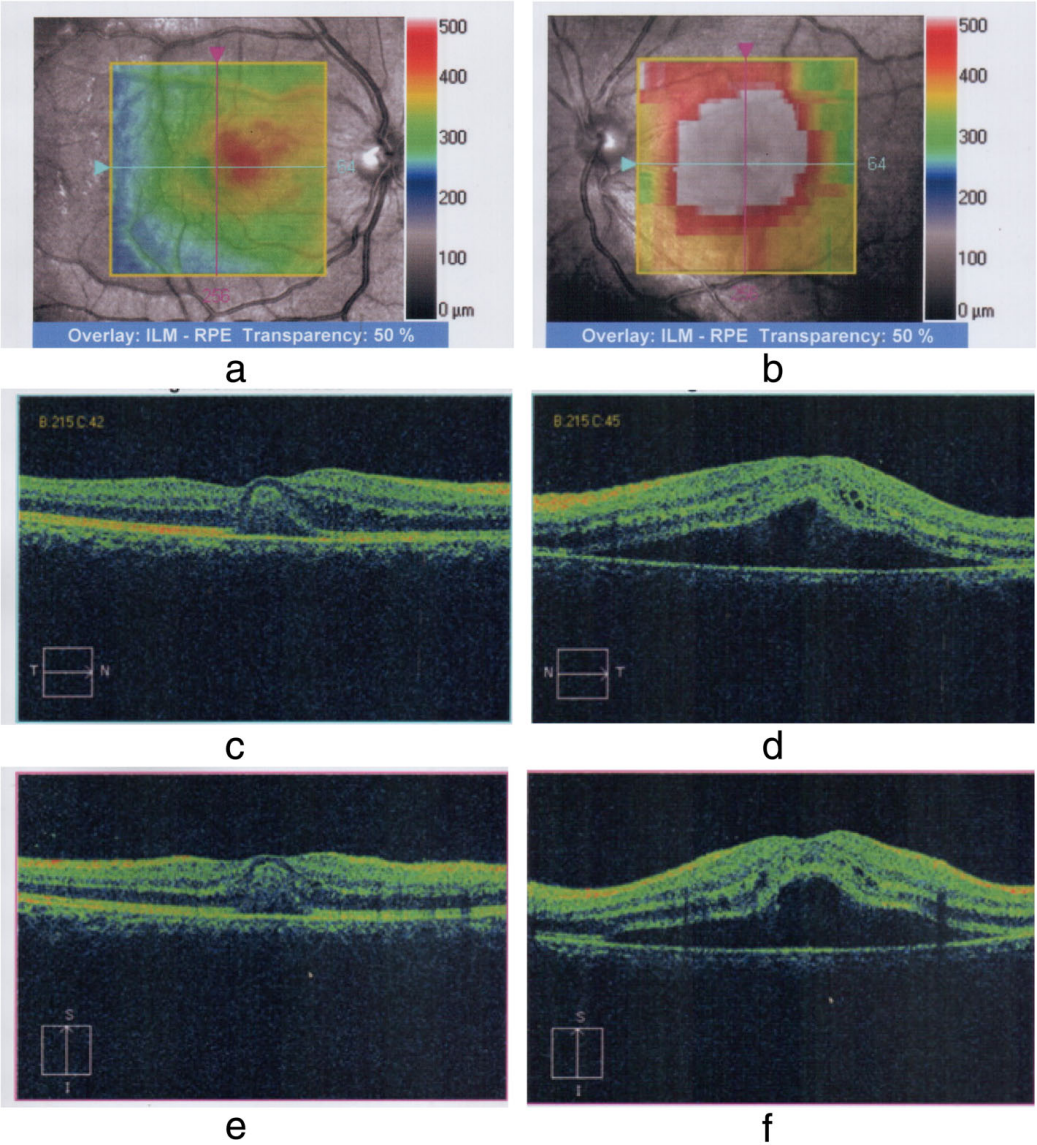
**a** and **b** Optical coherence tomography (OCT) of both eyes demonstrates retinal region involved. **a** Right eye. Increased thickness in the foveal region is demonstrated by the orange to pink hue. **b** Left eye. More marked involvement with a greater area affected is demonstrated by the pink and reddish hue centered on the fovea. Normal retinal thickness is < 320 μm within the green color spectrum, and the foveal thickness is usually 250 μm. **c**–**f** Macular scans concentrating on the region described in the OCT scans. **c** and **d** Horizontal cuts. **e** and **f** Vertical cuts. **c** and **e** Right eye. **d** and **f** Left eye. In both the horizontal (**d**) and vertical (**f**) cuts of the left eye, the foveal depression is lost and instead is elevated. The retinal pigmental epithelial  layer is lifted up, but not just at the fovea. Almost the whole macula is lifted up. The gap below the RPE is dark, suggesting the presence of fluid. This is a typical appearance of “central serous choroidoretinopathy.” In the right eye, depicted in **c** and **e**, elevation (second line from the bottom) is seen at the level of the fovea with a reflective material filling the space, suggestive of foveolitis

Our patient was a 27-year-old South Asian woman working as an intern medical officer. She is a teetotaller and nonsmoker, who was otherwise previously healthy, and has no significant family or social history of medical relevance. She presented with fever of 2 days’ duration associated with arthralgia and myalgia, for which she had taken only acetaminophen 1 g on an as-needed basis. On initial evaluation, she was febrile to touch, with a temperature of 100.6 °F. Her blood pressure on admission was 110 mmHg systole and 70 mmHg diastole, with a pulse rate of 96 beats per minute. A thorough general and systemic examination failed to elicit any other significant findings. DF was suspected and confirmed with a positive NS1 (nonstructural protein 1) antigen test on the second day. She was managed in accordance with national guidelines with precise fluid replacement, both orally and intravenously, with 0.9% normal saline. In addition to fluids, the only other medication administered was acetaminophen 1 g as needed based on her febrile state, which was stopped upon defervescence on day 4. Her vital signs and clinical indicators of perfusion (for example, pulse rate, blood pressure, capillary refill time, and urine output) were monitored and remained within acceptable reference ranges. Her initial complete blood count (CBC) on admission revealed hemoglobin (Hb) of 12.8 g/dl (normal range, 12–17.5) with hematocrit (HCT) of 34.4% (36–50%) and white blood cell count (WBC) of 4.59 × 10^9^/L (4–11 × 10^9^/L), a predominant neutrophilic differential of 83% (40–75%), and an initial platelet count of 186 × 10^9^/L (150–450 × 10^9^/L) (Table 1). Her CBC and HCT were monitored 6-hourly. On the second day, her baseline liver function tests revealed aspartate aminotransferase (AST) of 51 U/L (normal range, 10–35) and alanine aminotransferase (ALT) of only 34 U/L (10–40), but she complained of abdominal pain and significant nausea. Repeated assessment of liver function on day 5 revealed markedly elevated liver enzymes with AST of 1215 U/L (normal range, 10–35) and ALT of 630 U/L (10–40), which rose on day 6 to 1872 U/L and 1145 U/L, respectively (Table 1). Her total bilirubin remained normal at 17 mmol/L (normal range, 5–21). Her international normalized ratio remained within reference range, measuring 1.09, with a normal prothrombin time of 13.3 seconds (normal range, 10–14). Her activated partial thromboplastin time, however, was mildly prolonged at 45.4 seconds (normal range, 24–38) on day 6, rising to 57.6 seconds on day 7 (Table 1). An electrolyte profile was carried out and did not revealed gross abnormality. Her ionized calcium level was normal at 1.14 mmol/L (normal range, 1.12–1.32), serum potassium was 3.8 mmol/L (3.5–5.1), and sodium was mildly low at 133 mmol/L (135–148). Though her platelet counts declined, her packed cell volume and hemoglobin remained within acceptable stable parameters and together with her urine output did not indicate the onset of the leakage phase or suggest occult hemorrhage. On day 7, whole-blood analysis demonstrated Hb of 14.2 g/dl (normal range, 11–16) with HCT of 39% (37–54) and WBC of 3.35 × 10^9^/L (4–10 × 10^9^/L). The lowest documented platelet count was 27 × 10^9^/L (150–450 × 10^9^/L) (Table 1). Despite this, her clinical and other hematological parameters remained normal. The result of urinalysis was normal. On the eighth day of illness, upon waking up in the morning, the patient complained of blurred vision in both eyes. A bedside assessment revealed that the left eye detected only gross movements and the right eye had a visual acuity (VA) of 6/9. An urgent ophthalmology consult was obtained. Ocular examination with dilated funduscopy and fluorescein angiography (Fig. 1a–d) showed that the patient had subretinal fluid collections at the macular region with retinal nerve fiber layer hemorrhages in the papulomacular bundle area in the left eye and areas of hemorrhage in the right eye as well. The right eye also showed mild subretinal fluid at the macula. Optical coherence tomography (OCT) and macular scans (Fig. 2a–f) were used to assess the patient’s baseline status, which showed increased retinal thickness centering on the foveal region (more severe in the left eye) and elevation of the retinal pigment epithelial layer with collection of fluid with changes compatible with central serous choroid retinopathy in the left eye and foveolitis in the right eye (Fig. 2a–f). Repeat liver function tests on day 8 showed declining liver enzyme values (Table 1). Incidentally, her whole-blood analysis showed rising counts with WBC of 3.77 × 10^9^/L (normal range, 4–10 × 10^9^/L) and platelet value of 34 × 10^9^/L (150–450 × 10^9^/L) (Table 1), heralding recovery. A focused ultrasonographic study failed to reveal evidence of fluid leakage in the thoracic or abdominal cavities. However, her visual deficit remained. As treatment for the eye manifestations, she received a single 2-mg intravitreal triamcinolone injection into the right eye and 3 days of intravenous methylprednisolone 1 g following an ophthalmology consult.

**Table 1:** Blood cell counts and liver enzyme levels during hospital stay

	Day 2	Day 5	Day 6	Day 7	Day 8	Day 9	Day 12
Whole-blood analysis							
White cell count (reference range 4–11 × 10^9^)	4.59			3.35		3.77	18
Platelet count (reference range 150–450 × 10^9^)	186			27		34	336
Liver function							
Aspartate aminotransferase (reference range 10–35 U/L)	51	1215	1872		1020	531	
Alanine aminotransferase (reference range 10–40 U/L)	34	630	1145		805	617	
Activated partial thromboplastin time (reference range 28–34 seconds)			45.4	57.6

**Fig. 3:**
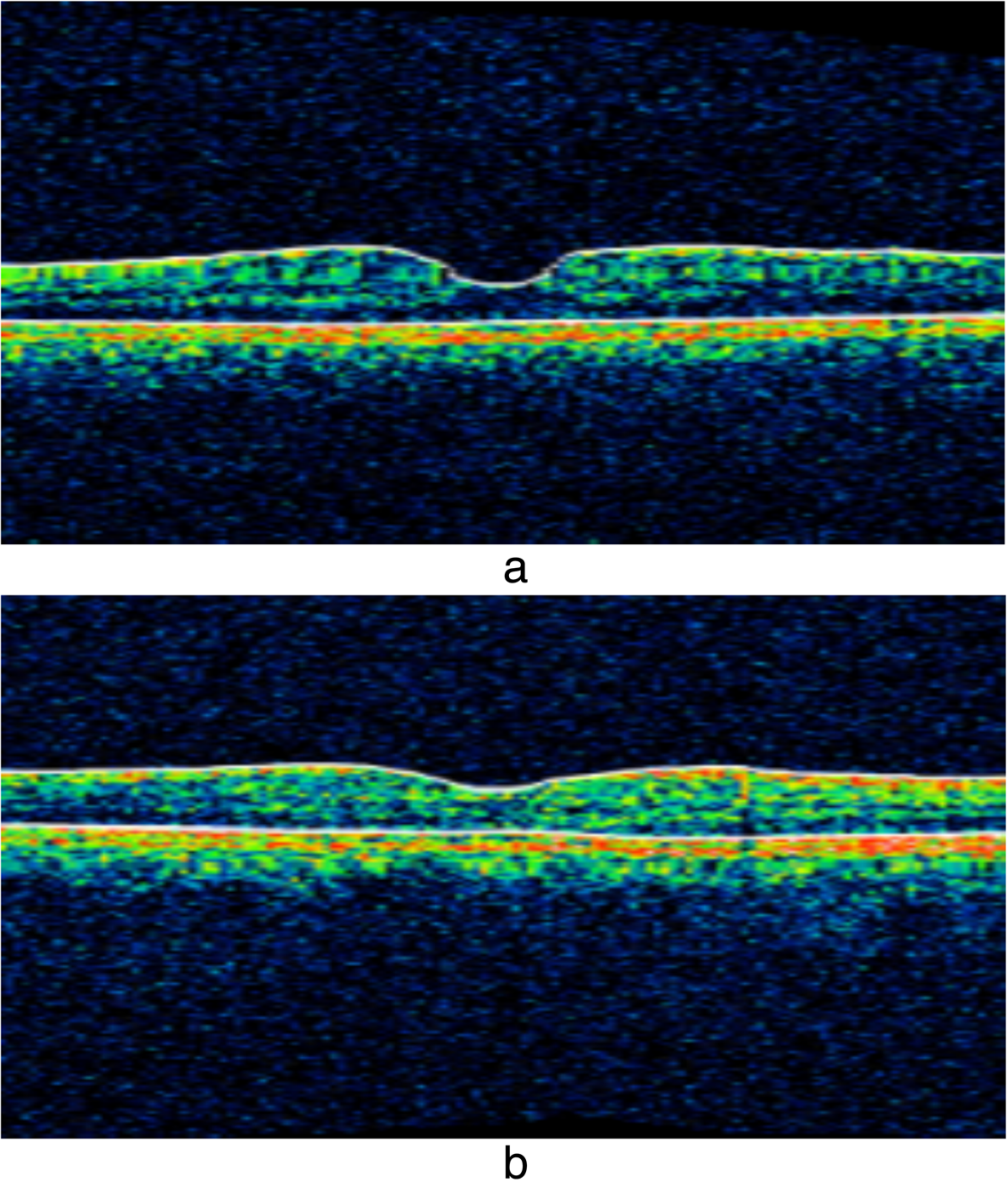
**a** and **b** Macula scans of the right and left eyes, respectively. Marked improvement is seen in both eyes compared with prior images of the same described in Fig. 2c and d

Upon discharge from the hospital on day 12, she had a platelet count of 336 × 10^9^ (normal range, 150–450 × 10^9^) with Hb of 12 g/dl (12–17.5) and an elevated WBC of 18 × 10^9^ (4–11 × 10^9^) (Table 1). Her elevated WBC was attributed to treatment with steroids because she was clinically normal with no fever and normal inflammatory markers: Her C-reactive protein was 0.4 mg/dl (< 8), and her erythrocyte sedimentation rate was 22 mm for the first hour. Ophthalmological reassessment prior to discharge revealed reduced but persisting macular edema and subretinal fluid collection in the eye. In follow-up, her VA was assessed frequently along with funduscopic examinations. Gradual recovery was observed. Despite normalization of her VA in her right eye, VA in her left eye remained at 6/9 at the end of 6 months. Similarly, assessment with OCT at 6 months to the day of initial assessment also demonstrated improvement from the previously seen changes (Fig. 3a, b). When she was reviewed 1 year later, her VA remained unchanged with persistence of 6/9 in the left eye.

## Discussion

This case report highlights the less frequently seen but nevertheless important complications that can occur with dengue viral infection. Ocular complications of dengue viral fever are quite uncommon, and when a combination of complications is seen, such as foveolitis and central serous chorioretinitis (CSCR) with profound visual impact, the role of active treatment vs. passive approach becomes questionable, especially when strict guidance is unavailable. In this case report, we attempt to give some insight into the etiopathogenesis, clinical picture, progression, investigations, and treatment options available following ocular involvement in dengue viral fever, and we describe the decisions we made for the ocular complications we encountered based on available evidence, in addition to the patient’s outcome.

The exact etiopathogenesis resulting in eye involvement in dengue is not clearly defined in the literature. Multiple hypotheses have been put forth, including low platelet counts predisposing to bleeding, and leakage due to increased permeability mediated by proinflammatory cytokines aided by coexistent inflammation as an immune-mediated hypothesis has also been suggested. Antibodies may also play a role with specific antibodies targeting various tissues of the eye. Furthermore, the altered pathogenicity and virulence of the disease based on serovar or mutation and the susceptibility of the host based on the mounted immune response might vary depending whether it is a primary or secondary infection, which may contribute to the observed extensive spectrum of the clinical picture [5, 6]. The usual timeline of eye involvement is seen in close association with the lowest platelet count values in both DF and DHF, a phenomenon observed by Chan *et al.* [7] and others, just as it occurred in our patient, and this reinforces the suspected platelet hypothesis, which suggests that complications in dengue virus infection occur at the lowest platelet count. However, Tan *et al.* [8] stated that this time frame also favors the immune-mediated hypothesis because it coincides with the production of immunoglobulin G, when the clinical picture heralds recovery signaling close association with the body’s immune response. None of the many suggested hypotheses, when considered in isolation, fully explains the spectrum of manifestations visualized in ocular involvement in dengue. This subject is still under study and is beyond the scope of this article.

Involvement of the eye, when it occurs, is usually bilateral, though it can also present unilaterally. The commonest symptom is blurring of vision; other symptoms include ocular pain, redness, metamorphopsia, impaired color vision, diplopia, eye flashes and floaters, haloes, and photophobia [4, 5, 9, 10]. The involvement of the macula results in the patient being symptomatic, but peripheral retinal involvement, such as retinal hemorrhage, may not be obvious and may be missed by the unaware clinician and the nonsymptomatic patient, implying that the true incidence of eye involvement in dengue may be underreported [11].

Observed signs on examination are vast and include hemorrhage, which can be sub-conjunctival or retinal; reduced VA; scotomas; inflammatory maculopathy with chorioretinitis; possible macular edema; and foveolitis. Vasculitis is also seen either focally or even in a panretinal distribution. Other signs include exudative retinal detachment, perifoveal telangiectasia, anterior uveitis, cotton wool spots, optic disc swelling, hyperemia, and neuritis [5, 7, 8, 12–14]. Uncommon occurrences include CSCR, which has been observed in dengue [15]. These manifestations can at times occur for the first time, months after the recovery of an otherwise uncomplicated dengue viral infection, as noted by Gupta *et al*. in their case series in which uveitis was seen as a delayed phenomenon, stressing the need for vigilance [6].

Funduscopic evaluation of the eye will help identify obvious retinal changes, such as retinal hemorrhage, cotton wool spots, and optic disc swelling. However, advanced investigations such as OCT have been found to be very useful, especially to define macular involvement and assess retinal thickness and morphology. Sometimes, infrared fundus photography (IFG) can shed more light and better delineate suspicious lesions which were appreciated on funduscopy that were not elucidated by OCT, because they can appear as dark patches in the retinal territory in the IFG report. The Amsler grid chart, a basic tool used to assess the visual field, can also help further delineate the scotomas, and this can be better appreciated by using an automated Humphrey field analyzer, which provides a comprehensive visual field assessment. Angiography with fluorescein or indocyanine green can further aid in identifying vascular lesions such as occlusion, leakage, and vasculitis [9, 16].

The definitive management is still disputed. Conservative management with close observation and follow-up is usually acceptable and has shown that recovery can occur unaided with complete resolution [7, 8]. In severe eye involvement such as that in our patient, however, active treatment has been chosen. Glucocorticoid therapy has been given as intravenous pulses, tapered oral regimens, or topical instillations [7], or even as intravitreal or sub-Tenon triamcinolone injections [6]. Immunosuppressive therapy with steroids has been shown to confer favorable overall outcomes with marked improvement, albeit with some residual deficit [9]. Intravenous immunoglobulin when intensive steroid therapy has failed or with worsening clinical picture has also been trialed and has been shown to be beneficial with improved VA and outcome [17, 18]. Thus, the positive response seen following immunosuppressive treatment possibly supports the immune-mediated hypothesis in ophthalmic manifestations of DF. However, the outcomes seen following immunosuppressive therapy, though encouraging, were not always uniform in their response. Furthermore, the strength of evidence is anecdotal at best, because most are based on case series and case reports, and none of the modalities of management are fully endorsed in either national or international guidelines. Intervention beyond conservative medical management has also been required with pars plana vitrectomy and panretinal photocoagulation for worsening hemorrhage and iridotomy for glaucoma [9]. The duration of recovery ranges from a few days to a few months. Full recovery has been observed. Similarly, residual deficit has also been noted, ranging from persistent mild central scotoma to impaired VA.

Our patient had the classic presentation with the onset of manifestation indicating eye involvement occurring at the lowest recorded platelet counts with a typical repertoire of symptoms and signs that are recognized when the eye is involved in dengue viral infection. The typical timeline stresses the need for heightened vigilance in this period and to expect ophthalmic complications when conducting bedside visual field assessment or acuity testing, which may aid in early detection and even identify asymptomatic patients.

Although our patient did not develop DHF, she did develop hepatitis along with minor derangement in her coagulation profile. Wills *et al*. observed that though coagulation derangement is a known phenomenon complicating dengue, even the presence of thrombocytopenia by itself does not contribute to hemorrhage unless complicated by shock and hypoxia with acidosis predisposing to disseminated intravascular coagulation precipitating bleeding [19]. Thus, the signs observed are not directly attributable to impaired coagulation and instead depend on the complex mechanisms discussed above. Our patient’s manifestations were uncommon, with foveolitis in the right eye and central serous chorioretinopathy in the left eye along with hemorrhages in both.

Central serous chorioretinopathy is a poorly understood phenomenon and more so when it occurs in dengue viral infection. The potential etiologies for CSCR include steroids, either exogenous or endogenous in origin; stress; psychological makeup; pregnancy; and genetic risk [20, 21]. CSCR has also been observed in cases of autoimmune disease and infections [22, 23]. In pregnancy, one of the postulated causes is the increased permeability [21]; though the mechanism is different, a similar increase in permeability is appreciated in the spectrum of dengue viral infection. Similarly, dengue viral fever can cause significant stress and a subsequent rise in catecholamine levels [15]. One could postulate that these are potential mechanisms for CSCR to occur in patients with dengue viral infection in addition to the patient factors such as psychological makeup. Taking this into consideration, the challenge we faced was to determine the treatment strategy for our patient: whether aggressive treatment was to be chosen or to let the disease run its course and await spontaneous resolution. Our patient developed foveolitis and CSCR due to dengue viral infection. The clinical problem we faced was that foveolitis is known to benefit from steroids, whereas CSCR may potentially worsen with steroid therapy [24, 25]. Though evidence favoring treatment is anecdotal, owing to the severity of the presentation, a combined clinical decision was made to opt for systemic glucocorticoid therapy. The clinical response was favorable; the patient was able to engage in daily activities with very little in the way of limitation due to a visual deficit, which can be elucidated only with Amsler charting. However, whether the observed improvements were either fully or partially attributable to the treatment is speculative. The clinical problem of choosing not to treat or choosing a less intensive treatment is a difficult decision, considering the risk of permanent visual deficit. Thus, with lack of clearly defined guidelines, the decision should be at the discretion of the clinical team and the patient.

## Conclusion

Involvement of the eye in dengue viral infection, though uncommon, should garner a greater awareness and appreciation. Any visual complaint should be regarded as potential dengue eye involvement, especially around the time platelet counts drop to their lowest value and begin to rise. To understand the true incidence of dengue eye involvement and the patterns of presentation, as well as to formulate a clear protocol for management, further focused research is necessary.

## Data Availability

All the data used and/or analyzed during case report development are included in this case report.

## Abbreviations

ALT: Alanine aminotransferase
AST: Aspartate aminotransferase
CBC: Complete blood count
CSCR: Central serous chorioretinitis
DF: Dengue fever
DHF: Dengue hemorrhagic fever
Hb: Hemoglobin
HCT: Hematocrit
IFG: Infrared fundus photography
OCT: Optical coherence tomography
VA: Visual acuity
WBC: White blood cell count
